# Analysis of risk factors for complications after femoral neck fracture in pediatric patients

**DOI:** 10.1186/s13018-020-01587-9

**Published:** 2020-02-19

**Authors:** Zhen-Zhen Dai, Zhi-Qiang Zhang, Jing Ding, Zhen-Kai Wu, Xuan Yang, Zi-Ming Zhang, Hai Li

**Affiliations:** 1 Department of Pediatric Orthopedics, Xin Hua Hospital Affiliated to Shanghai Jiao Tong University School of Medicine, 1665 Kongjiang Road, Yangpu District, Shanghai, 200092 China; 2 Department of Pediatric Orthopedics, National Children’s Medical center & Children’s Hospital of Fudan University, 379 Wanyuan Road, Minhang District, Shanghai, China

**Keywords:** Femoral neck fracture, Pediatric, Risk factors, Complications

## Abstract

**Background:**

Pediatric femoral neck fracture is a rare injury but yields frequent complications. However, there is a paucity of data regarding the risk factors for these complications.

**Purpose:**

The present article reports the rate of complications after femoral neck fracture in pediatric patients and investigates the possible risk factors.

**Methods:**

We retrospectively reviewed 44 children (mean age of 9.0 years, range from 2 to 14 years) who were surgically treated for femoral neck fracture in a single trauma center with a mean follow-up of 57.75 months (range from 11 to 224 months). Related clinical factors were recorded and analyzed by multivariable logistic regression.

**Results:**

Fracture displacement or Delbet-type fracture had no relation to the injury mechanism. However, younger children experienced severe trauma, combined injury, and low fracture location more than older individuals did. Children with combined injuries were more likely to have a longer waiting time for surgical reduction. Common complications included avascular necrosis (AVN) in 14 cases, nonunion of fracture in 2 cases, coxa vara in 4 cases, and premature physeal closure (PPC) in 7 cases. Only the Delbet type was an independent predictor of AVN (OR = 0.14, *p* = 0.030). Inadequate reduction was associated with higher rates of coxa vara (OR = 33.19, *p* = 0.032). Epiphysis penetration in children younger than 10 years old increased the rate of PPC (*p* = 0.032). No significant risk factor was found for fracture nonunion.

**Conclusion:**

For femoral neck fracture in pediatric patients, both the injury mechanism and fracture characteristics have age-related distributions. Early reduction should be carried out as early as possible based on the safe condition of the child, but for younger children, transepiphyseal fixation should be avoided. AVN may be intrinsic to injury characteristics rather than resulting from the choice of treatment mode.

## Background

Femoral neck fracture is a rare injury in pediatric patients, accounting for approximately 1% of all fractures in childhood [1, 2] but yields relatively frequent surgical complications, such as avascular necrosis (AVN) of the femoral head, nonunion of fracture, coxa vara, and premature physeal closure (PPC) [3–5]. These complications may be attributed to the open physis in the proximal femur and fragile structure of blood support for the femoral head in pediatric patients [6, 7] and could lead to antalgic gait, permanent limping, and limited range of motion [3, 4, 8]. Among these complications, AVN has been confirmed as a significant risk for hip arthritis, but thus far, there is no effective treatment [4]. Other complications, such as nonunion of fracture, coxa vara, and PPC, sometimes require repeated surgery [8]. However, some factors related to trauma or the treatment mode may be associated with complications. Therefore, understanding and reducing these risks is of great significance for surgeons to guide treatment, decrease complications, and improve the prognosis of rare injuries. The primary purpose in the present retrospective research was to review the rate of complications after femoral neck fracture in pediatric patients and to investigate the possible risk factors.

## Patients and methods

After institutional review board (IRB) approval, we retrospectively reviewed all of the patients treated for femoral neck fracture in the Department of Pediatric Orthopedics at our hospital from 2006 to 2016. The inclusion criteria were as follows: (1) surgical treatment within 3 weeks of injury, (2) growth plate of the proximal femur, which has growth potential at the time of trauma, and (3) minimum follow-up of 11 months with complete medical data. Children with pathological bone fractures and those with cerebral palsy or metabolic bone disease were excluded.

Generally, in our institution, children with femoral neck fracture were urgently admitted and evaluated for their status, such as whether this condition was combined with life-threatening injuries. As soon as the status of the patient was stable, surgical reduction and fixation were performed.

Close or open reduction was determined by the senior surgeon depending on the displacement of the fracture. For open reduction, the lateral patient position and the Watson-Jones approach were usually used, and capsular decompression was performed. Based on the location of the fracture, lag screws or proximal hip plates (PHPs) were implanted to maintain the fracture reduction. Whether the implant screws penetrated the proximal femur epiphysis depended on the reduction stability during the operation.

Postoperatively, all patients were immobilized by spica casting or similar orthosis until there was confirmation of the callus crossing of the fracture site in radiological images evaluated by two senior physicians. Gradual weight-bearing, restoration of range of motion, close follow-up, and regular radiological examinations were applied to all patients. Hardware removal was performed, from at least 6 months to 1 year postoperatively, if solid bone union was obtained.

Clinical factors related to trauma, fracture and operation, surgical outcome, and complications were recorded from chart and radiographic reviews, which were performed by two senior physicians, as follows: (1) preoperative information: demographic information, mechanisms of injury, combined injury, fracture types, and displacement; (2) operative information: time from injury to reduction, types of reduction, internal fixation, quality of reduction, and whether the implant screws penetrated the proximal femur epiphysis; (3) surgical outcomes scored according to Ratliff’s criteria [9] and complications (AVN, nonunion of fracture, coxa vara, and PPC).

The mechanism of injury was categorized as follows: (1) sports-related injury, (2) fall injury, and (3) motor vehicle accident. Fracture types were based on the modified Delbet type reviewed in plain radiographs, as follows: type I (transepiphyseal), type II (transcervical), and type III (cervicotrochanteric) [10, 11]. Delbet type IV (intertrochanteric fractures) was not included in this study.

The quality of reduction postoperatively was evaluated according to fracture alignment as the modified radiographic criteria and scored as good and not good. Good alignment was defined as < 4-mm step-off and < 5-degree angulation [12]. The surgical outcome was evaluated by Ratliff’s criteria. All of the complications were determined by reviewing plain radiographs or magnetic resonance images (MRIs). The postoperative time until the diagnosis of AVN was also recorded. If no or minimal healing was seen in plain radiographs for more than 3 months, the diagnosis of nonunion was established. Coxa vara was defined as neck-shaft angles of < 120°. Both the radiographs and surgical outcomes were reviewed by three senior surgeons.

The correlation between clinical factors was assessed by the Fisher exact test or Wilcoxon test for categorical variables and by linear regression or the *t* test and analysis of variance (ANOVA) for continuous variables. Analysis of risk factors for complications was evaluated by logistic regression models, and odds ratios (ORs) with their 95% confidence intervals (CIs) were also obtained. Factors included in the multivariate model were those with *p* < 0.5 in the univariable analysis or those found as significant predictive factors in the literature [13–16]. Statistical tests were considered significant at *p* < 0.05. All analyses were performed with the statistical software Stata version 14.0.

## Results

### Injury characters

Forty-four patients were included in our study (27 boys and 17 girls, mean age 9.0 years old, range from 2 to 14 years old). Sports-related injury was the most common mechanism of fracture (*n* = 26, 59%) (Table 1). Combined injuries were only related to car accidents (*n* = 6) and falls from a height (*n* = 4), which was confirmed in 10 children (23%) (Table 1), including 6 brain injuries, 9 pulmonary contusions, 8 multiple fractures (pelvic, femoral, or tibial fracture), and 1 spleen injury.

**Table 1:** Demographics and treatment characters

Variable	Study cohort (*n* = 44)
Sex (no. [%])	
Male	27 (61%)
Female	17 (39%)
Age* (years) [range]	9.0 ± 3.68 [2–14]
Injury mechanism (no. [%])	
Sports-related injury	26 (59%)
Fall from height	10 (23%)
Car accident	8 (18%)
Combined injury (no. [%])	10 (23%)
Displaced fracture (no. [%])	37 (84%)
Delbet type (no. [%])	
I	1 (2%)
II	25 (57%)
III	18 (41%)
Time to reduction* (days) [range]	4.27 ± 3.76 [1–19]
Early reduction (within 24 h after injury) (no. [%])	7 (16%)
Type of reduction (no. [%])	
Close reduction	13 (30%)
Open reduction with capsular decompression	31 (70%)
Good reduction quality (no. [%])	40 (91%)
Type of internal fixation (no. [%])	
Lag screws	31 (70%)
Plate and screws	13 (30%)
Epiphysis penetration (no. [%])	7 (16%)

*The value is presented as the mean ± SD

Eighty-four percent of the fractures were displaced fractures, and more than half of the fractures (*n* = 25, 57%) were Delbet-type II fractures (Table 1). However, fracture displacement had no association with the Delbet type of fracture, and neither of classification had any relation to the injury mechanism.

Both injury mechanism and fracture characteristics had an age-related distribution, with younger children experiencing severe trauma (10.69 ± 3.08 years vs. 7.6 ± 3.27 years vs. 5.25 ± 2.43 years, *p* = 0.0001), combined injury (10.24 ± 3.06 years vs. 4.8 ± 2.25 years, p = 0.0001), and a low fracture location (Delbet type III) (9.85 ± 3.46 years vs. 7.78 ± 3.73 years, *p* = 0.0660) more than older individuals did.

### Operative characters

The mean time from injury to surgical reduction was 4.27 days (range from 1 to 19 days) (Table 1), of which 16% (*n* = 7) was within 1 day and 41% (*n* = 18) was within 2 days. From the multiple linear regression analysis, we found that a longer waiting time to reduction was more likely to occur in younger children with combined injury or severe trauma, and if adjusted for other factors, only combined injury (adjusted coef., 6.46; CI, 3.76–9.16, *p* = 0.000) was an independent risk for a longer waiting time to surgical reduction. Open reduction with capsular decompression was performed on 31 children (70%), and good reduction quality was obtained in 40 children (91%) (Table 1).

### Operative outcomes and complications

The mean follow-up time for surgical outcome and complications was 57.75 months (range from 11 to 224 months). The surgical outcome by Ratliff’s criteria showed that 66% of children exhibited grade 1, 32% presented grade 2, and 2% showed grade 3. The common postoperative complications included AVN in 14 children (32%), nonunion of fracture in 2 children (5%), coxa vara in 4 children (9%), and PPC in 7 children (16%). The time to diagnosis of AVN ranged from 7 to 20 months postoperatively (average time, 11.79 months). Moreover, in children without early reduction (37 children later than 1 day and 26 children later than 2 days), the incidence of AVN was 35.1% (13/37) and 34.6% (9/2), respectively. Fortunately, the child who underwent reduction after 19 days did not develop AVN postoperatively.

### Risks for complications

For AVN, Delbet type and epiphysis penetration were found to be significant risk factors in the univariate analysis (Table 2), while adjusted for other factors, only the Delbet type was an independent risk factor for developing AVN (adjusted OR, 0.14; CI, 0.03–0.83, *p* = 0.030) (Table 3). Furthermore, by stratified analysis, early surgical reduction did not reduce the risk of AVN for children with Delbet I or II fractures.

**Table 2:** Patient and fracture characteristics by occurrence of complications

Characteristics	*n* (%)* or mean ± SD (range)									
	AVN (*n* = 14)	No. AVN (*n* = 30)	*p*	Cox vara (*n* = 4)	No. vara (*n* = 40)	*p*	Nonunion (*n* = 2)	*p*	Preclosure (*n* = 7)	*p*
Sex			1.000			1.000		1.000		1.000
Female	5 (36)	12 (40)		1 (25)	16 (40)		1 (50)		3 (43)	
Male	9 (64)	18 (60)		3 (75)	24 (60)		1 (50)		4 (57)	
Age	9.57 ± 3.16 (5–14)	8.73 ± 3.92 (2–14)	0.454	8.50 ± 3.70 (5–13)	9.05 ± 3.72 (2–14)	0.792	11.5 ± 2.12 (10–13)	0.303	9.43 ± 2.99 (6–13)	0.701
Injury mechanism			0.817			1.000		1.000		0.613
Sports-related injury	8 (57)	18 (60)		2 (50)	24 (60)		2 (100)		3 (43)	
Fall from height	4 (29)	6 (20)		1 (25)	9 (23)		0 (0)		2 (29)	
Car accident	2 (14)	6 (20)		1 (25)	7 (18)		0 (0)		2 (29)	
Delbet type			**0.019**			0.662		1.000		0.335
I	1 (7)	0 (0)		0 (0)	1 (3)		0 (0)		0 (0)	
II	11 (79)	14 (47)		3 (75)	22 (55)		1 (50)		6 (86)	
III	2 (14)	16 (53)		1 (25)	17 (43)		1 (50)		1 (14)	
Displaced fracture	13 (93)	24 (80)	0.401	4 (100)	33 (83)	1.000	2 (100)	1.000	6 (86)	1.000
Combined injury	3 (21)	9 (26)	1.000	2 (50)	8 (20)	0.218	0 (0)	1.000	2 (29)	0.649
Surgical reduction within 24 h after injury	1 (7)	6 (20)	0.401	0 (0)	7 (18)	1.000	0 (0)	1.000	0 (0)	0.575
Type of reduction			1.000			0.302		1.000		0.654
Close reduction	4 (29)	9 (30)		0 (0)	13 (33)		0 (0)		1 (14)	
Open reduction with capsular decompression	10 (71)	21 (70)		4 (100)	27 (68)		2 (100)		6 (86)	
Fixation			1.000			1.000		0.508		1.000
Lag screws	10 (71)	21 (70)		3 (75)	28 (70)		1 (50)		5 (71)	
Plate and screws	4 (29)	9 (30)		1 (25)	12 (30)		1 (50)		2 (29)	
Pass epiphysis	5 (36)	2 (7)	**0.025**	1 (25)	6 (15)	0.513	0 (0)	1.000	3 (43)	0.068
Good Reduction quality	13 (93)	27 (90)	1.000	2 (50)	38 (95)	**0.036**	1 (50)	0.175	7 (100)	1.000

Bold values are statistically significant *p* < 0.05

*Patient number with the percentage of patient number in total patient of the column within parentheses

**Table 3:** Odds ratio estimates for risk factors of complications

Complications	Risk factors	Odds ratio	(95% CI*)	*p*
AVN	Displaced fracture	4.78	(0.43–52.69)	0.201
	Delbet type	0.14	(0.03–0.83)	**0.030**
	Early reduction	0.91	(0.72–1.16)	0.448
	Pass epiphysis	2.67	(0.36–19.54)	0.333
Coxa vara	Delbet	0.30	(0.03–3.50)	0.335
	Combined injury	6.04	(0.14–257.59)	0.347
	Early reduction	1.04	(0.67–1.61)	0.858
	Reduction quality	33.19	(1.35–814.53)	**0.032**
Preclosure	Age	0.98	(0.76–1.27)	0.889
	Pass epiphysis	4.73	(0.70–31.89)	0.111
	Delbet	0.45	(0.07–3.14)	0.422
Nonunion	Delbet	1.36	(0.07–27.88)	0.841
	Age	1.20	(0.69–2.09)	0.511
	Reduction quality	8.59	(0.31–239.58)	0.205

*CI indicates confidence interval

Bold values are statistically significant *p* < 0.05

For coxa vara, reduction quality was the independent risk factor, and if adjusted for other factors, poor reduction increased approximately 34 times the possibility of developing coxa vara (adjusted OR, 33.19; CI, 1.35–814.53, *p* = 0.032) (Tables 2 and 3).

In addition, screws penetrating the epiphysis were not found to be a significant risk factor for PPC (Tables 2 and 3), whereas the stratification analysis revealed that, for children younger than 10 years old, epiphysis penetration (compared with no epiphysis penetration) increased the possibility of developing PPC (*p* = 0.032). Furthermore, no significant risk factor was found for nonunion of fracture (Tables 2 and 3).

## Discussion

The fracture line in pediatric femoral neck fracture is usually uniplanar without interlocking like that in adults, which makes these pediatric fractures highly unstable [7]. Compared with conservative treatment, operative treatment with internal fixation yields fewer postoperative complications and better outcomes and has been accepted by most surgeons [16, 17]. Therefore, in our series, surgical treatment with internal fixation was used for each case, even in fractures without displacement [18, 19], whereas the association of conservative treatment with the rate of complications was not evaluated.

In addition, femoral neck fracture is rare in pediatrics, and our institution is a pediatric trauma center in eastern China. Approximately 87% of children were transferred from other hospitals or cities, and this might be the reason that only 16% of children underwent early reduction (within 1 day after injury) in our institution. After admission, approximately 41% of pediatric patients underwent reduction within 2 days to ensure the safety of children with severe combined injury or unstable circulatory conditions. As the study showed, combined injury was the only independent factor in deferring the time to surgical reduction. This means that early reduction cannot be entirely controlled by the surgeon and patients.

The rate of AVN (32%) in our study was similar to that reported in Spence’s study (29%) [13] but higher than that in Yeranosian’s meta-analysis (23%) [16]. However, the time to diagnosis of AVN was as long as 20 months, while the shortest follow-up time was 11 months in our series, which means that the real rate of AVN may be underestimated. In our study, only the Delbet type was shown to be an independent risk factor for AVN, which was partly consistent with the results in the literature [13–16]. However, neither time from injury to reduction nor fracture displacement was an independent risk factor for AVN. In particular, for Delbet-type I/II fractures, early surgical reduction did not decrease the risk of developing AVN, and displaced fracture did not increase the risk of developing AVN. Therefore, the development of AVN may occur as an independent entity with relation to injury characteristics rather than the mode of treatment carried out.

The prevalence of coxa vara was 9% in the present study and 17% in the literature [16]. Reduction quality, rather than fracture displacement or Delbet type, was found to be an independent risk factor for coxa vara, which could be relatively controlled by the surgeon. In the literature [15, 16], operative treatment with internal fixation for femoral neck fracture was found to be related to a lower rate of coxa vara. In our series, all of the children underwent surgical reduction with internal fixation, while the association of conservative treatment with the rate of coxa vara was not evaluated. Coxa vara is a known risk factor for hip arthritis [3, 4, 8] and should be avoided by good fracture reduction as far as possible, both conservatively and surgically.

The incidence of PPC has been reported to be 20–30% after femoral neck fracture and was 16% in our series [15, 16]. We know that the proximal femoral physis contributes approximately 15% of the overall length of the femur and that coxa vara and coxa valga deformities of the proximal femur may result from partial premature physeal arrest [20]. There is a debate about whether screws passing the epiphysis are related to PPC because the epiphyseal plate remains open after penetration by screws in some children but closed without penetration in others [3, 21]. We did not find that screws passing the epiphysis were related to PPC. We only used screws passing the epiphysis for Delbet type I and II fractures because we consider stable reduction more important than epiphysis closure during surgical reduction. In contrast, for children younger than 10 years old, epiphysis penetration greatly increased the possibility of developing PPC. PPC did have a strong relation to lower extremity discrepancy [15], so for young children, surgeons should be cautious in pursuing stable reduction and should weigh the pros and cons.

The rate of fracture nonunion was lower in our research (5%) than that (8%) in the literature [15]. However, no risk factor was found to be predictive for nonunion of fracture. The primary cause of nonunion is a failure to obtain or maintain anatomic reduction [20], and in our series, both internal fixation and postoperative immobilization were used for each child. Despite such rigorous treatment rules, there were two children with nonunion of fracture. We attributed this outcome to loss of reduction. It was difficult to evaluate the stability of screws, especially after repeatedly implanting the screws during the procedure, which may have impaired the stability of surgical reduction.

The present study has several limitations. First, the intrinsic limits of a retrospective study in a single center cannot be avoided completely. Second, the limited number of patients included may weaken the strength of results from multivariate analyses. However, femoral neck fracture in pediatric practice is rare, and the results of the present study are mostly consistent with those of other published investigations. A multicenter prospective study may be a better way of studying these rare but challenging injuries.

## Conclusions

Generally, in order to reduce the incidence of complications, surgeons should perform urgent reduction based on the safe condition of the child, should try their best to achieve anatomical reduction but should be cautious about epiphyseal penetration in young children. Moreover, surgeons should inform parents that AVN is related to the characteristic of the fracture (Delbet type) rather than the choice of surgical procedure.

## Data Availability

The datasets used and analyzed during the current study are available from the corresponding author on reasonable request.

## Abbreviations

AVN: Avascular necrosis
PPC: Premature physeal closure
